# Supplementary material for: Are carbon emissions trading and green financial instruments synergistic? -Comprehensive quantitative research based on content analysis
Source: PLoS One. 2024 Mar 7;19(3):e0298601. doi: 10.1371/journal.pone.0298601 (PMC10919601; doi:10.1371/journal.pone.0298601)
Supplement: S1 Data — (ZIP) [file pone.0298601.s001.zip › Data/地级市Z_F工作报告.zip!/╘╞─╧/└Ñ├≈╩╨/└┤╫╘╣1⁄2╓┌║┼í╛┬φ┐╦╩2╛▌═°í┐_2015.docx]

各位代表：

　　现在，我代表市人民政府向大会报告工作，请予审议，并请市政协委员提出意见。

　　一、2014年工作回顾

　　过去的一年，面对复杂严峻的国内外形势和艰巨繁重的改革发展稳定任务，在省委、省政府和市委的坚强领导下，在市人大及其常委会和市政协的监督支持下，市政府坚持稳中求进，全力以赴稳增长、促改革、调结构、惠民生、保稳定，推动了经济社会发展稳中有进。预计全市地区生产总值3710亿元、增长8%，不含滇中产业新区为3280亿元、增长8.2%；地方公共财政预算收入444亿元，增长8.4%；规模以上固定资产投资3138亿元、增长7%，不含滇中产业新区为2670亿元、增长12%；社会消费品零售总额1900亿元，不含滇中产业新区为1800亿元，均增长12%；城镇、农村常住居民人均可支配收入预计分别突破3万元和1万元，分别增长9%和11%；居民消费价格上涨3.1%。

　　一年来，我们主要做了以下工作：

　　（一）狠抓结构调整转型升级，产业结构不断优化。规模以上工业增加值增长8%以上，高技术工业企业增加值占6%左右，规模以上工业固定资产投资完成604亿元，59个亿元以上工业项目竣工，园区规模以上工业增加值占全市的86%。服务业增加值增长7.5%，商贸服务业集聚区加快建设，螺蛳湾国际商贸城、斗南国际花卉产业园等重点片区初步形成，新增总部企业23户、楼宇面积220万平方米。马来亚银行、渣打银行在昆设立分行。旅游总收入609亿元，增长18%。农业增加值增长6.1%，新增省级龙头企业7户，都市农庄建设完成投资10亿元，初步建成12个。三次产业结构调整为5.0︰44.5︰50.5。

　　（二）注重功能完善统筹发展，城乡建设稳步推进。编制完成城市总体规划、历史文化名城保护规划等一批规划。以交通为重点的基础设施建设加快推进，南连接线建成通车，绕城高速内环线实现闭合；东南绕城开工，呈澄、黄马、功待高速顺利推进，全市“环线+射线”骨干路网基本形成。呈七公路建成通车，东倘、寻倘公路一期、金东大桥加快推进。地铁1、2号线首期工程开通运营，3号线加快建设，6号线二期及1号线呈贡支线顺利开工。铁路征地拆迁基本完成，昆明南站及配套工程全面推进。综合整治13条城市重要道路、6条微循环道路和14个交通节点，二环内实行货车限行。城乡公交运营线路达861条，主城公共交通出行分担率提高4.8个百分点、达52.1%。建制村公路路面硬化648公里，公交通达率95%。深入实施城乡清洁工程，开展重点区域专项整治，拆除临违建筑169万平方米，新建垃圾中转站10座。全面实施主城行洪河道清淤除障、管网疏通及节点改造，城市内涝问题得到有效缓解。分类推进“城中村”改造，新启动拆迁8个，交付使用回迁安置房210万平方米，安置拆迁户7000户。完成主城区29个商品交易市场整治搬迁。县域经济综合实力持续壮大，5个区进入省县域经济先进县行列。放宽县城和建制镇落户条件，农村居民转户11.8万人，城镇化率达69%。建成美丽乡村80个。实施农村危房改造和宜居农房建设2.76万户。完成水利投资31.3亿元，续建的3件中型水库、14件小（1）型水库加快建设，实施46件小（2）型病险水库除险加固，建成5万件“五小水利”工程，解决21万农村人口饮水安全问题。加快实施扶贫攻坚三年行动计划，整合筹集资金16.4亿元，贫困人口减少6.8万人。

　　（三）全面深化改革扩大开放，发展活力逐步增强。放宽注册资本条件，除特定行业企业外，实行注册资本认缴登记制。实行工商登记“先照后证”制度，新登记企业同比增长62.1%。农村集体建设用地使用权和宅基地发证工作取得成效，全市流转农村土地102.5万亩，占家庭承包土地面积的43.7%。价格改革稳步推进，制定了主城区管道天然气临时价格，实施农业用水终端水价制度。深化考试招生制度改革，出台了“小升初”改革方案和异地中考实施方案。食品药品监管体制改革顺利推进，建立了覆盖市县乡的监管体系。鼓励引导社会资本参与公共领域的建设营运，首批80个基础设施项目向社会投资开放。民营经济预计实现增加值1700亿元，增长8%。科技创新能力进一步提升，新认定市级产业技术创新战略联盟3个、重点实验室和工程技术研究中心25个，全市专利授权量达4526件。实际利用外资16.7亿美元，引进市外到位资金995亿元。实现进出口贸易总额177.9亿美元。成功举办第二届中国—南亚博览会暨第22届昆交会，签订招商引资项目82个。昆明泛亚国际农业博览会升级为省级博览会。综合保税区申报、招商、建设取得阶段性成果。“一县一市一街道”托管移交滇中产业新区。72小时过境免签政策落地实施。荣获“国际友好城市交流合作奖”，新缔结国际友城1个。

　　（四）强化环境保护资源节约，生态建设取得实效。滇池治理完成投资61.1亿元，污水处理厂尾水外排及资源化利用等治污工程建成运行。滇池水质持续改善，化学需氧量、总氮、总磷指标分别下降5.63%、10.3%、14.2%，国家考核的16条河道有14条水质达标。城市污水日处理能力达197万立方米，出水水质全面达到一级A标。牛栏江—滇池补水工程投入运行，实现生态补水4亿多立方米。启动“国家生态园林城市”创建工作，主城建成区新增绿地1200公顷。完成人工造林42.8万亩，森林覆盖率达49%。完成基本农田划定工作，1.8万亩低丘缓坡土地综合开发利用试点项目获批。建成国家生态乡镇5个和省级生态文明县区5个。完成省下达的重点减排项目110个，预计万元生产总值能耗下降4%左右。主城空气质量优良率达97%。

　　（五）坚持以人为本改善民生，社会事业协调发展。全面落实“十件惠民实事”，深入实施“春风化雨”行动，民生支出375.5亿元，占地方公共财政预算支出的69%。多渠道多形式促进就业创业，提供有效就业岗位15.8万个，城镇新增就业11.8万人，城镇登记失业率2.27%，农村劳动力转移就业17.2万人。建成国家级创业孵化示范基地1个、省市级大学生创业示范园15个。新建、改造标准化菜市场、生鲜超市61个，建成10个商业服务示范社区、5条特色旅游文化商业街区。落实社保标准与物价上涨联动机制，城市低保、农村低保和五保供养标准分别提高15.38%、19.5%和16.2%。城乡社会保险参保覆盖面进一步扩大，城乡居民医疗保险一体化建设顺利推进。养老服务体系不断完善，建设社区居家养老服务中心67个，新增养老床位4737张。开工建设保障性住房、棚户区改造住房30121套，建成3.3万套，完成保障性住房分配、租赁住房补贴发放9996套（户）。推进教育均衡发展，学前教育毛入园率提升到97.3%，义务教育巩固率保持在99%以上，高中阶段毛入学率达92%以上。安排各类资金7.1亿元，资助贫困家庭幼儿和学生99.8万人次。农村义务教育阶段学校营养改善计划惠及35.9万名学生。完成校舍改造工程27.9万平方米。完成37个村卫生室标准化建设，市第一人民医院北市区医院、市第三人民医院烈性传染病诊疗中心投入使用，全市新增病床3210张。“单独两孩”政策稳步实施，人口自然增长率5.58‰。公共文化服务体系日趋完善，新建、改扩建文化站（室）73个，“两馆一站”全部免费开放，举办各类文化惠民演出活动1700多场，实施不可移动文物保护工程33项。建成文化体育活动广场51个，全面完成市体育学校暨全民健身中心建设，成功举办昆明环滇池高原自行车邀请赛。果断处置“3·01”暴力恐怖案件。晋宁“10·22”事件和“10·14”事件处置工作取得阶段性成效。妥善处置“9·26”明通小学踩踏事件、“10·28”东川山体滑坡地质灾害，重点行业、重点场所的安全生产监管得到加强。

　　（六）切实转变作风提升效能，政府自身建设得到加强。扎实开展党的群众路线教育实践活动，严格落实中央“八项规定”，认真整改“四风”方面存在的突出问题，“三公”经费下降4.1%。认真执行市人大及其常委会决议决定，坚持重大事项报告制度，自觉接受市人大及其常委会的法律监督、工作监督，主动接受市政协的民主监督，认真办理人大代表建议和政协委员提案。提请市人大常委会制定、修订地方性法规4件，制定、修订政府规章和规范性文件7件，重大决策公示、听证417件。承接省级下放行政审批事项105项，取消和下放市级行政审批事项16项。公共资源实现电子化交易，成为国家公共资源电子化交易创新试点城市。严格执行党风廉政建设责任制，健全廉政风险防控体系，加强行政监察和审计监督，严肃查办了一批违法违纪案件。外事侨务、民族宗教、妇女儿童、残疾人、档案、气象、防震减灾、人民防空、双拥等工作取得新的进步。

　　各位代表！回顾过去的一年，我们深感成绩来之不易。这些成绩的取得，是省委、省政府和市委正确领导的结果，是市人大、市政协、社会各界支持帮助和全市人民奋力拼搏的结果。在此，我代表市政府，向全市各族人民，向人大代表、政协委员，向各民主党派、工商联、人民团体和社会各界人士，向中央省属单位、各兄弟州市，向驻昆解放军和武警官兵，向关心支持昆明建设发展的海内外朋友，表示崇高敬意和诚挚感谢！

　　各位代表！回顾过去的一年，我们深感压力巨大、充满艰辛。由于我们对经济发展“新常态”认识不足，对经济形势的严峻性和复杂性估计不充分，特别是在经济下行压力持续加大的情况下，我市长期存在的深层次结构性矛盾凸显，经济增长支撑乏力，加之发展环境不优、主观努力不够，导致经济增长低于预期，地区生产总值、固定资产投资、地方财政收入和城乡常住居民人均可支配收入等指标的增长没有完成既定目标。与此同时，全市经济社会发展中还存在着一些不容忽视的问题，主要表现在：一是经济总量不大、质量不优，持续发展的动力不足。二是城乡区域发展差距较大，发展不平衡、不协调问题突出。三是城市规划的前瞻性、科学性、严肃性不够，建设缺乏统筹谋划，管理水平不高。四是就业、教育、医疗、住房等方面存在许多薄弱环节，安全生产、食品药品安全等方面的问题依然较多。五是资源环境约束加剧，滇池治理任重道远。六是推进改革的方法不多、力度不大，重要领域和关键环节的改革推进不快。七是社会治理法治化水平不高，社会矛盾多发易发、交错叠加。八是少数干部能力不足、精神懈怠、缺乏担当、脱离群众等问题较为突出，腐败现象时有发生。对于这些问题，我们将高度重视，切实加以解决。
